# Association of migration status with quality of life among rural and urban adults with rare diseases: A cross-sectional study from China

**DOI:** 10.3389/fpubh.2022.1030828

**Published:** 2022-11-10

**Authors:** Huanyu Zhang, Shanquan Chen, Dong Dong

**Affiliations:** ^1^Shenzhen Research Institute, The Chinese University of Hong Kong, Shenzhen, China; ^2^School of Clinical Medicine, University of Cambridge, Cambridge, United Kingdom; ^3^JC School of Public Health and Primary Care, Faculty of Medicine, The Chinese University of Hong Kong, Sha Tin, Hong Kong SAR, China

**Keywords:** migration, rare diseases, quality of life, rural, urban

## Abstract

**Background:**

A considerable proportion of rare disease patients decide to migrate to access a definitive diagnosis or appropriate care, which could affect their quality of life in a long term.

**Objective:**

To compare quality of life (QoL) between migrants and residents and explore the possible mechanism of how migration influence the QoL among rural and urban adults with rare diseases, respectively.

**Methods:**

A cross-sectional study at national level was conducted in a study sample of 1,150 adult patients in China. Migration was defined as being away from one's original place of residence for at least 12 months. Patients who remained in their place of residence in the past 12 months (“resident”) were treated as a comparison group for “migrants”. Original area of residence (rural vs. urban) for both residents and migrants was used for comparison. The brief version of the World Health Organization Quality of Life instrument was used to measure QoL. Multiple linear regression analyses were adopted to assess the direct association between migration status and QoL after controlling for the confounders that affect QoL. The indirect associations between migration status and QoL, mediated by potential mediators including number of family members living together, individual income, catastrophic health expenditure, and social support, were estimated using the mediation model.

**Results:**

Among the group of rural participants, migration was directly associated with physical QoL (β = 5.07, 95% CI 2.01–8.13) and environmental QoL (3.95, 1.37–6.53), indirectly associated with physical QoL (0.58, 0.05–1.28) and social QoL (0.50, 0.01–1.16) *via* individual income, and also indirectly associated with environmental QoL (−0.47, −1.12 to −0.50) *via* tangible support. On the other hand, neither direct nor indirect associations of migration with four domain scores of QoL were significant among the group of urban participants.

**Conclusion:**

Among rural adults with rare diseases, migration was found to have positive direct effect on physical and environmental QoL, positive indirect effect on physical and social QoL through increased individual income, and negative indirect effect on environmental QoL *via* reduced tangible support. By contrast, neither direct nor indirect associations of migration with QoL were significant among the group of urban participants.

## Background

Rare diseases affect 6–8% of the global population (1, 2). While each rare disease represents unique experience, individuals with rare diseases could have some common issues, one of which is difficulty in accessing an accurate diagnosis. Based on the results of a survey published in 2013, it took 7.6 years in the United States and 5.6 years in the United Kingdom for a patient with a rare disease to receive an accurate diagnosis, with an average of two to three misdiagnoses along the way (3). Misdiagnoses or diagnostic delays can cause the worsening of clinical status of rare disease patients, thus leading to inefficient medical treatments and additional health costs (4, 5).

Another common issue that rare disease patients could experience is barriers to appropriate care. There are a limited number of specialists, who have expertise in a given rare disease nationally or even globally. In addition, lack of awareness and knowledge with rare diseases among health professionals leads to inability to provide appropriate referral recommendations (6–8).

Due to difficulties in accessing a definitive diagnosis or appropriate treatment, patients with rare diseases have been traveling long distances to access proper medical care. Even, a considerable proportion of individuals has to relocate to access medical care related to their rare disease on a permanent or longer-term basis. A survey conducted by the National Organization for Rare Disorders in 2019 revealed that 17% of the 1,108 investigated adults, who were affected by a rare disease or the caregiver or a family member of someone with a rare disease, have already relocated or were considering it in the United States (9). A survey conducted in Europe showed that 16% of the 5,995 involved patients affected by rare diseases had to move house motivated by their disease-related needs (10).

Migration is deemed as an important factor affecting health-related quality of life (HRQoL). Previous studies have revealed that compared with individuals of the host population, migrants generally enjoy better quality of life (QoL) related to the increase of income and better services and resources in the migration destination at early stages of migration, however, this advantage tends to diminish over time (11, 12). Nonetheless, selecting those native to the host communities as a comparison group might not allow us to differentiate the effects of migration on QoL from pre-existing health and socioeconomic disparities between the often poor sending locales and the more developed receiving locales (13, 14). Hence, the more appropriate approach is to compare the QoL of migrants with those who remain in the sending locale for they share similar attributes. In China, QoL of migrants is closely related to the household registration system (*hukou* in Chinese), which is very difficult to transfer from rural to urban areas (13, 15). Compared with rural residents, urban residents usually enjoy better public welfare and social services in terms of education, housing, healthcare, and retirement benefits (12, 13). Due to the differences in access to health and social services between rural and urban residents, it is of interest to look into the impact of migration on QoL among these two populations, separately.

Unlike the referral systems in many other countries, patients in mainland China can directly access healthcare services in any hospital without a referral recommendation. This encourages rare disease patients to migrate to urban areas with high-quality healthcare in China (16, 17). Although this healthcare-seeking process may or may not be covered by patients' medical insurance, the coverage of medical insurance for the care of rare disease patients in China is quite limited (18). Furthermore, a previous study conducted by our research team showed that the coverage of medical insurance had no significant effect on trans-provincial diagnosis in China (17). Therefore, whether having insurance coverage from the government only played a limited role in affecting the willingness of rare disease patients to migrate in China, and thus was not considered in the current study.

Previous studies have shown that the effect of migration on QoL was complicated through multiple immediate and offsetting pathways, with some factors beneficial and others being harmful (13, 14). Nonetheless, few studies have focused on the influence of migration on QoL among rare disease patients at population level to the best of our knowledge. Clear evidence and possible mechanisms are of great significance for the development of customized interventions for rural and urban migrants with rare diseases, separately. Therefore, in this study, we hypothesized that disparities existed between rural and urban populations on the association between migration and QoL. Our primary aim is to compare the HRQoL between migrants and residents among adults with rare diseases, with a separate attention to rural and urban populations. Our second aim is to explore the possible mechanism of how migration influence the QoL among adults with rare diseases.

## Methods

### Study sample and data collection

**Figure 1:**
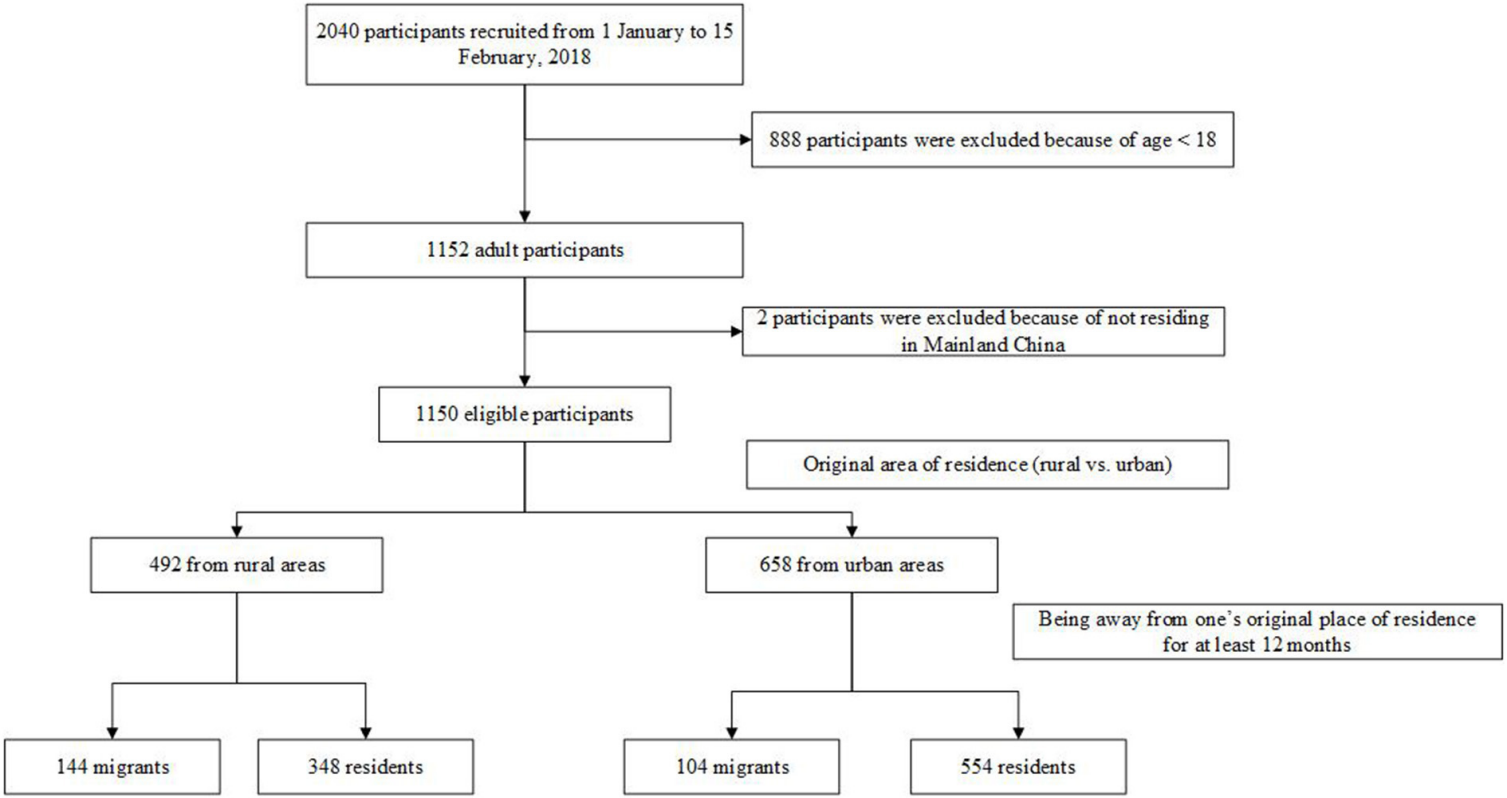
Construction of the study sample.

A cross-sectional study at national level was conducted from 1 January through 15 February, 2018. Since the epidemiological information of patients affected by rare diseases in China is absent, a non-probability convenience sampling method was employed to recruit participants in collaboration with Illness Challenge Foundation, a national umbrella organization working together with 29 multiple rare disease patient organizations in China. With the support of the Foundation's network, an online survey was used to reach a population of rare disease patients as widely as possible across the country. Before the survey was officially conducted, we'd invited representatives from patient organizations as well as experts in the areas of medicine and social science to review the structure and contents of the questionnaire. A pilot study was also conducted in a small sample of rare disease patients to examine if they could understand each question. When the online survey was formally distributed among the study sample, participants followed a link to the questionnaire website and must click “consent to participate” button before they could access the complete questionnaire. In total, 2,040 valid responses were collected, from which 1,152 were adults (aged 18 years and older). We excluded two adults for they had not resided in Mainland China. Finally, a sample of 1,150 adult patients, affected by 75 different rare diseases across 31 provinces in Mainland China, was included in the current study. The construction of the study sample was presented in Figure 1.

### Measurements

#### Migration status assessment

The minimum migration time have not been consistently defined in prior studies conducted in China (14, 19). In this study, to examine the effect of migration on health in a relatively longer term, being away from one's original place of residence for at least 12 months was defined as migration. Correspondingly, the remained residents were treated as a comparison group for migrants. To differentiate the effects of migration on health from the pre-existing health disparities between the rural and urban populations, both migrants and their comparisons were defined within their original area of residence (rural vs. urban) (Figure 1).

#### Demographic and socioeconomic characteristics

We investigated the following sociodemographic and economic characteristics, including age, gender (male vs. female), marital status (married or cohabiting vs. others), number of family members living together, education (primary school or lower, middle school, high school, 3-year college, and bachelor or above), employment (unemployed, employed, and no working capacity), and annual individual income (per 1,000 RMB).

Considering the potential predictors of QoL among people with rare diseases and their families (18, 20, 21), we also investigated the disease economic burden (yes or no, measured by catastrophic health expenditure [CHE]), dependence on assistive devices (yes or no), and social support. CHE was a commonly used indicator to measure the economic burden. The criterion for defining CHE is not commonly accepted, which varies from 10% of family income (22), 10% of household consumption (23), to 40% of disposable income (24, 25). In this study, we selected the criterion for measuring CHE as out-of-pocket health expenditures in excess of 10% of annual family income (22). The reason why we choose this criterion is because it is less sensitive to individuals' behavior of saving and consuming compared with the other two criteria, and thus more adapted to the Chinese context. In China, as a precaution, people are more inclined to save money by avoiding unnecessary consumptions, while they are relatively generous when they seek medical help for their loved ones (26, 27). Therefore, we used the 10% of family income as the criterion for CHE in the current study. Information on dependence on assistive devices (yes or no) was collected by asking the degree to which the participants needed to rely on assistive devices in their daily lives with five responses including “don't need at all, occasionally need, sometimes need, often need, and completely need”. The latter four responses were coded as “yes”. Social support was measured by the Chinese Mandarin version of the Medical Outcomes Study Social Support Survey (MOS-SSS-CM) (28). The 19-item MOS-SSS-CM comprises four subscales, i.e., 4-item tangible support, 8-item emotional/ informational and support, 4-item positive social interaction, and 3-item affectionate support. The scores of four subscales are transformed to a standardized 0–100 scale, with higher scores indicating better perceived support (29).

#### Quality of life

The brief version of the World Health Organization Quality of Life (WHOQOL-BREF) instrument was used to measure QoL in this study. The WHOQOL-BREF is a multi-dimensional instrument to assess individuals' perceptions of their goals, expectations, standards, and concerns in the context of specific culture and value systems (30). The Chinese translation of WHOQOL-BREF has been widely used to measure QoL in multiple populations, including rare disease patients (18, 31). The instrument is constructed by the following four domains, namely, physical (7 items), psychological (6 items), environmental (8 items), and social relationships (3 items). The physical domain is represented by pain, energy, sleep, mobility, daily activities, medical treatment, and work. The psychological domain is constituted by positive/negative feelings, concentration, self-esteem, bodily appearance, and spirituality. The environment domain refers to physical safety and security, living conditions, finance, access to health/social services, leisure, physical environment, and transportation. The domain of social relationships emphasizes patients' personal relationships, sex activities, and support from friends. Each domain score is transformed to a scale of 0–100 according to the guideline published by the WHO, with higher scores denoting higher levels of QoL (30).

### Analytical strategies

Descriptive analyses were conducted between migrants and local residents among rural and urban participants, respectively. Continuous variables were reported as medians (interquartile ranges [IQRs]) and tested by Wilcoxon test. Categorical variables were reported as frequencies (percentages) and tested by Fisher's Exact test.

To assess the direct association between migration status and QoL, we adopted multiple linear regression analyses, by the four dimensions of QoL and original area of residence, controlling for age, gender, marital status, family number, education, annual individual income, CHE, dependence on assistive devices, and social support (Equation 1). Where *Y*_*QoL*_ is physical QoL, psychological QoL, environmental QoL, or social QoL; *migration* is a binary variable (yes or no) indicating the migration status of an individual; *x*_1_+…+ *x*_*n*_ are the covariates controlled. Since the variable of employment is associated with annual individual income, to avoid multicollinearity, employment was not included in the multiple linear regression models.


(1) $$\begin{array}{l} Y_{QoL}=\;\alpha +\;\beta \;*\;migration\;+\;\gamma_{1}\;*\;x_{1}\\ \;+\ldots +\;\gamma_{n}\;*\;x_{n}+\;\varepsilon \end{array}$$

To further control for possible unbalances between migrants and residents, the models fitted by Equation 1 were adjusted by inverse probability weights (IPWs). Inverse probability weighting is an extension of the propensity score method used to handle the unbalance among intervention groups (32). We derived IPWs from propensity scores generated by a logistic regression with migration status as the outcome, controlling for the same covariates included in the Equation 1.

We further estimated the indirect association between migration status and QoL, also by the four dimensions of QoL and original area of residence. To do this, we firstly conducted a series of regressions between migration status and possible mediators (Equation 2). Where *Y*_*Mediator*_ is a possible mediator; *migration* is a binary variable (yes or no) indicating the migration status of an individual; *x*_1_+…+ *x*_*n*_ are the covariates controlled.


(2) $$\begin{array}{l} Y_{Mediator}=\;\alpha +\;\beta \;*\;migration\;+\;\gamma_{1}\;*\;x_{1}\\ \;+\ldots +\;\gamma_{n}\;*\;x_{n}+\;\varepsilon \end{array}$$

**Figure 2:**
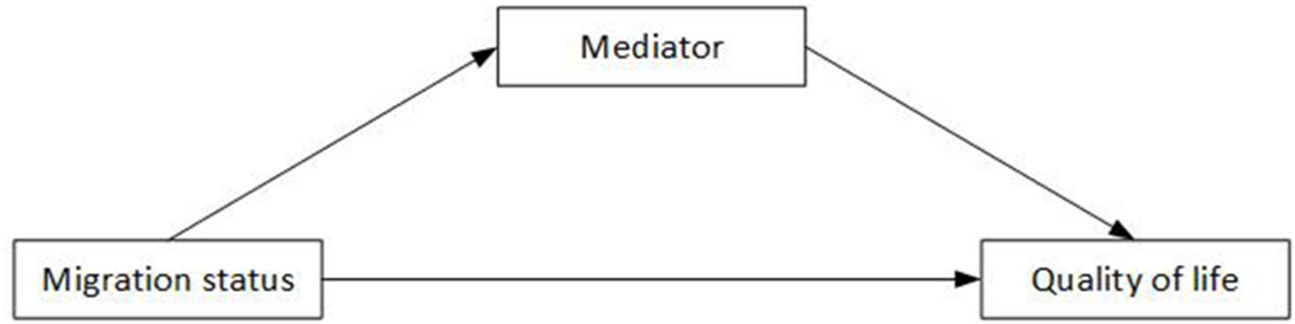
Mediation model in estimating the indirect association between migration status and QoL.

The mediators that we selected in our datasets may have potential implications for future interventions on improving QoL of migrants, including the number of family members living together, individual income, CHE, and social support. For the family number, individual income, and social support, Equation 2 were fitted by linear regressions, controlling for the remaining factors. For CHE, Equation 2 were fitted by logistic regression, controlling for the remaining factors. Secondly, the mediation model (Figure 2) was used to estimate the indirect association based on the results from the regression between migration status and QoL (Equation 1) and regression between migration status and possible mediator (Equation 2). Only mediators that had significant association with both QoL and migration status were further included in the mediation model. Mediation model in Figure 2 was implemented by function “mediate” in package mediation (version 4.5.0) in R [Ref “mediation: R Package for Causal Mediation Analysis”].

All statistical analyses were performed using the R software (version 4.0.4). Statistical significance was defined as *p* < 0.05.

## Results

Of the 1,150 participants included, 492 (42.8%) originally lived in rural areas, and 537 (46.7%) were males, with a median age of 34.0 (IQR 28.0–43.0). Nearly 60% of the study sample were married or cohabiting. Only 39.8% of the participants were employed, and 45.2% had to depend on assistive devices. More detailed information on demographics of the participants is shown in Table 1.

**Table 1:** Descriptive characteristics of urban and rural adults with rare diseases by migration status.

	**Total**	**Rural**, ***n*** = **492**		***p*-value**	**Urban**, ***n*** = **658**		***p*-value**
	***N* = 1,150**	**Migrants**,	**Resident**,		**Migrants**,	**Resident**,	
		***n* = 144**	***n* = 348**		***n* = 104**	***n* = 554**	
Age	34.0 (28.0–43.0)	31.0 (26.0–37.0)	32.0 (28.0–39.0)	**0.023**	32.0 (27.8–40.0)	37.0 (30.0–46.0)	**0.002**
**Gender**				0.092			0.747
Male	537 (46.7%)	81 (56.3%)	165 (47.4%)		44 (42.3%)	247 (44.6%)	
Female	613 (53.3%)	63 (43.8%)	183 (52.6%)		60 (57.7%)	307 (55.4%)	
**Married/Cohabiting**				0.093			0.123
No	485 (42.2%)	77 (53.5%)	157 (45.1%)		47 (45.2%)	204 (36.8%)	
Yes	665 (57.8%)	67 (46.5%)	191 (54.9%)		57 (54.8%)	350 (63.2%)	
**Number of family members living together**	3 (2–4)	3 (2–4)	4 (3–5)	**0.028**	3 (2–4)	3 (2–4)	0.164
**Education**				**<0.001**			**0.010**
Primary school or lower	141 (12.3%)	20 (13.9%)	72 (20.7%)		4 (3.8%)	45 (8.1%)	
Middle school	253 (22.0%)	37 (25.7%)	136 (39.1%)		11 (10.6%)	69 (12.5%)	
High school	274 (23.8%)	39 (27.1%)	85 (24.4%)		18 (17.3%)	132 (23.8%)	
3-year college	203 (17.7%)	23 (16.0%)	33 (9.5%)		18 (17.3%)	129 (23.3%)	
Bachelor or above	279 (24.3%)	25 (17.4%)	22 (6.3%)		53 (51.0%)	179 (32.3%)	
**Employment**				**<0.001**			0.747
Unemployed	490 (42.6%)	63 (43.8%)	175 (50.3%)		41 (39.4%)	211 (38.1%)	
Employed	458 (39.8%)	64 (44.4%)	81 (23.3%)		51 (49.0%)	262 (47.3%)	
No working capacity	202 (17.6%)	17 (11.8%)	92 (26.4%)		12 (11.5%)	81 (14.6%)	
**Individual income (per 1,000 RMB)**	14 (0–40)	20 (0–40)	2 (0–20)	**<0.001**	20 (0–60)	20 (0–50)	0.859
**Catastrophic health expenditure**				0.280			**0.025**
No	365 (31.7%)	49 (34.0%)	102 (29.3%)		42 (40.4%)	172 (31.0%)	
Yes	750 (65.2%)	89 (61.8%)	234 (67.2%)		54 (51.9%)	373 (67.3%)	
Missing	35 (3.0%)	6 (4.2%)	12 (3.4%)		8 (7.7%)	9 (1.6%)	
**Dependence on assistive devices**				**<0.001**			**0.024**
No	630 (54.8%)	95 (66.0%)	166 (47.7%)		69 (66.3%)	300 (54.2%)	
Yes	520 (45.2%)	49 (33.0%)	182 (52.3%)		35 (33.7%)	254 (45.8%)	
**Social support**							
Tangible support	65.0 (50.0–80.0)	60.0 (45.0–75.0)	65.0 (50.0–80.0)	0.075	65.0 (50.0–80.0)	70.0 (55.0–85.0)	**0.029**
Emotional/informational support	55.0 (40.6–67.5)	51.2 (40.0–62.5)	47.5 (37.5–62.5)	0.100	57.5 (47.5–70.0)	57.5 (45.0–70.0)	0.924
Positive social interaction	55.0 (40.0–65.0)	55.0 (40.0–65.0)	45.0 (35.0–60.0)	**0.011**	57.5 (45.0–70.0)	55.0 (40.0–70.0)	0.842
Affectionate support	56.7 (40.0–73.3)	53.3 (40.0–66.7)	46.7 (33.3–60.0)	0.131	60.0 (46.7–73.3)	60.0 (46.7–73.3)	0.657

Data are reported as median (IQR) for continuous variables and number (percentage) for categorical variables. Bold values indicate statistical significance which is defined as p-value <0.05.

Of the 492 rural participants, 144 (20.3%) were designated as migrants, while this number was 104 (15.8%) among the 658 urban respondents in this study. For both groups of rural and urban participants, migrants were significantly younger (*p* = 0.023 vs. 0.002), had a higher level of education (*p* < 0.001 vs. *p* = 0.010), and less likely to depend on assistive devices compared with resident counterparts (*p* < 0.001 vs. *p* = 0.024). On the other hand, rural migrants had a significantly fewer number of family members living together (*p* = 0.028), more likely to be employed (*p* < 0.001), a higher individual income (*p* < 0.001), and more positive social interactions compared with rural residents (*p* = 0.011), whereas no significant differences were found in these aspects between urban migrants and residents (*p* > 0.05). In the group of urban participants, migrants were found to have a significantly higher probability of suffering from CHE (*p* = 0.025), and less likely to receive tangible support compared with resident counterparts (*p* = 0.029).

Table 2 presented four domain scores of QoL among urban and rural participants by migration status. Compared with resident counterparts, rural migrants had a significantly higher score in the domains of physical (57.1 vs. 42.9, *p* < 0.001), psychological (45.8 vs. 37.5, *p* = 0.001), and environmental QoL (43.8 vs. 35.9, *p* < 0.001), whereas no significant difference was found in the domain of social QoL (50.0 vs. 50.0, *p* = 0.07). By contrast, in the group of urban participants, the scores of physical (53.6 vs. 50.0, *p* = 0.064), psychological (45.8 vs. 45.8, *p* = 0.356), environmental (46.9 vs. 43.8, *p* = 0.266), and social QoL (50.0 vs. 50.0, *p* = 0.507) between migrants and residents were not significantly different.

**Table 2:** Quality of life scores among urban and rural adults with rare diseases by migration status.

	**Rural**, ***n*** = **492**		***p*-value**	**Urban**, ***n*** = **658**		***p*-value**
	**Migrants, *n* = 144**	**Residents, *n* = 348**		**Migrants, *n* = 104**	**Residents, *n* = 554**	
Physical QOL	57.1 (42.9–67.9)	42.9 (28.6–57.1)	**<0.001**	53.6 (39.3–64.3)	50.0 (32.1–64.3)	0.064
Psychological QOL	45.8 (33.3–58.3)	37.5 (25.0–50.0)	**0.001**	45.8 (33.3–58.3)	45.8 (29.2–58.3)	0.356
Environmental QOL	43.8 (33.6–53.1)	35.9 (25.0–46.9)	**<0.001**	46.9 (37.5–56.2)	43.8 (31.2–58.6)	0.266
Social QOL	50.0 (41.7–58.3)	50.0 (33.3–58.3)	0.070	50.0 (41.7–66.7)	50.0 (35.4–66.7)	0.507

Data are presented as median(IQR). Bold values indicate statistical significance which is defined as p-value <0.05.

After controlling for confounders, among the group of rural participants, migration was directly associated with physical QoL (β = 5.07, 95% CI 2.01–8.13) and environmental QoL (β = 3.95, 95% CI 1.37–6.53), while the direct associations between migration and psychological QoL (β = 3.16, 95% CI −0.20–6.53) and social QoL (β = 0.88, 95% CI −2.18–3.94) were not significant, as shown in Table 3. Furthermore, migration indirectly associated with physical QoL (β = 0.58, 95% CI 0.05–1.28) and social QoL (β = 0.50, 95% CI 0.01–1.16) *via* individual income, and also indirectly negatively associated with environmental QoL (β = −0.47, 95% CI −1.12 to −0.05) *via* tangible support, as shown in Table 4.

**Table 3:** Direct effects of migration on the four dimensions of QoL for rural adults with rare diseases.

**Variables**	**Physical QoL**	**Psychological QoL**	**Environmental QoL**	**Social QoL**
Migration status (Ref = No)	**5.07 (2.01, 8.13)**	3.16 (−0.20, 6.53)	**3.95 (1.37, 6.53)**	0.88 (−2.18, 3.94)
Age	−0.04 (−0.21, 0.13)	0.11 (−0.08, 0.30)	0.06 (−0.08, 0.20)	0.01 (−0.15, 0.18)
Gender (Ref = Male)	**3.35 (0.38, 6.31)**	−0.96 (−4.22, 2.30)	1.23 (−1.27, 3.73)	**7.31 (4.35, 10.28)**
Education (Ref = Primary school or lower)				
Middle school	−0.03 (−4.18, 4.13)	−0.07 **(**−4.64, 4.49)	0.73 (−2.77, 4.23)	0.10 (−4.05, 4.25)
High school	1.35 (−3.12, 5.82)	1.48 **(**−3.43, 6.39)	1.39 (−2.37, 5.16)	2.00 (−2.47, 6.47)
3-year college	3.12 (−2.30, 8.53)	1.40 **(**−4.55, 7.35)	3.99 (−0.57, 8.55)	1.78 (−3.63, 7.20)
Bachelor or above	−5.79 (−12.00, 0.42)	–**7.95 (**–**14.78**, –**1.13)**	−2.91 (−8.14, 2.31)	−5.64 (−11.85, 0.57)
Married/Cohabiting (Ref = No)	−3.00 (−6.48, 0.48)	–**5.76 (**–**9.59**, –**1.93)**	−1.52 (−4.45, 1.41)	−0.28 (−3.76, 3.20)
Family number	0.00 (−0.88, 0.89)	0.53 (−0.44, 1.50)	0.26 (−0.48, 1.01)	−0.03 (−0.91, 0.85)
Individual income	**0.09 (0.03, 0.16)**	0.04 (−0.03, 0.11)	0.02 (−0.04, 0.07)	**0.06 (0.00, 0.12)**
CHE (Ref = No)	–**6.01 (**–**9.07**, –**2.95)**	−2.55 (−5.91, 0.81)	–**3.04 (**–**5.61**, –**0.46)**	0.85 (−2.21, 3.90)
Dependence on assistive devices (Ref = No)	–**15.34 (**–**18.32**, –**12.36)**	–**3.22 (**–**6.49**, –**0.06)**	–**6.12 (**–**8.63**, –**3.61)**	–**3.10 (**–**6.08**, –**0.12)**
Social support				
Tangible support	−0.02 (−0.11, 0.07)	0.02 (−0.08, 0.12)	**0.10 (0.03, 0.18)**	−0.04 (−0.13, 0.05)
Emotional/informational support	0.09 (−0.08, 0.27)	0.02 (−0.17, 0.21)	**0.22 (0.07, 0.36)**	0.14 (−0.03, 0.31)
Positive social interaction	**0.40 (0.23, 0.56)**	**0.35 (0.17, 0.53)**	**0.20 (0.06, 0.35)**	**0.35 (0.18, 0.52)**
Affectionate support	−0.01 (−0.15, 0.13)	**0.23 (0.08, 0.39)**	0.04 (−0.08, 0.16)	**0.14 (0.00, 0.29)**

Data are presented as coefficients and their 95% confidence intervals. Bold values indicate statistical significance which is defined as p-value <0.05.

**Table 4:** Indirect effects of migration on the four dimensions of QoL for rural adults with rare diseases.

**Mediators**	**Migration status**	**Physical QoL**	**Psychological QoL**	**Environmental QoL**	**Social QoL**
Family number	−0.25 (−0.59, 0.10)	–	–	–	–
Individual income	**7.29 (3.00, 11.59)**	**0.58 (0.05, 1.28)**	–	–	**0.50 (0.01, 1.16)**
CHE	−0.01 (−0.46, 0.45)	–	–	–	–
Tangible support	–**4.89 (**–**8.05**, –**1.73)**	–	–	–**0.47 (**–**1.12**, –**0.05)**	–
Emotional/informational support	0.34 (−1.39, 2.07)	–	–	–	–
Positive social interaction	0.19 (−1.66, 2.04)	–	–	–	–
Affectionate support	1.06 (−1.05, 3.17)	–	–	–	–

Data are presented as coefficients and their 95% confidence intervals. Column 2 presents the results from “migration status – mediator”, controlling for other variables listed in Table 1. Column 3–6 presents the results from “migration status – mediator ~ corresponding QoL”, controlling for other variables listed in Table 1. “–” indicates that the potential mediators have no significant associations with the corresponding QoL or migration status has no significant associations with mediators. Bold values indicate statistical significance which is defined as p-value <0.05.

On the other hand, among the group of urban participants, neither direct nor indirect associations of migration with four domain scores of QoL were significant, as shown in Tables 5, 6.

**Table 5:** Direct effects of migration on the four dimensions of QoL for urban adults with rare diseases.

**Variables**	**Physical QoL**	**Psychological QoL**	**Environmental QoL**	**Social QoL**
Migration status (Ref = No)	−0.28 (−3.77, 3.20)	−0.19 (−3.73, 3.34)	−0.46 (−3.39, 2.47)	−0.81 (−4.04, 2.41)
Age	−0.06 (−0.20, 0.08)	−0.03 (−0.17, 0.11)	0.06 (−0.05, 0.18)	−0.02 (−0.15, 0.11)
Gender (Ref = Male)	–**2.81 (**–**5.48**, –**0.13)**	–**3.95 (**–**6.66**, –**1.25)**	−1.16 (−3.41, 1.09)	0.80 (−1.67, 3.28)
Education (Ref = Primary school or lower)				
Middle school	2.02 (−3.93, 7.96)	1.95 (−4.07, 7.97)	0.05 (−4.95, 5.05)	2.05 (−3.45, 7.55)
High school	1.36 (−4.06, 6.78)	3.32 (−2.17, 8.82)	1.66 (−2.90, 6.22)	2.94 (−2.08, 7.96)
3–year college	2.35 (−3.24, 7.94)	**5.46 (0.20, 11.12)**	4.56 (−0.14, 9.26)	4.99 (−0.19, 10.16)
Bachelor or above	4.10 (−1.37, 9.57)	**6.33 (0.79, 11.87)**	**5.00 (0.40, 9.60)**	**5.99 (0.92, 11.05)**
Married/Cohabiting (Ref = No)	−0.24 (−3.72, 3.23)	1.78 (−1.74, 5.30)	−0.51 (−3.43, 2.41)	**2.84 (0.37, 6.06)**
Family number	−0.16 (−1.21, 0.89)	−0.46 (−1.52, 0.60)	−0.72 (−1.60, 0.16)	−0.19 (−1.16, 0.78)
Individual income	**0.03 (0.00, 0.06)**	0.02 (−0.01, 0.06)	**0.04 (0.01, 0.06)**	**0.03 (0.00, 0.06)**
CHE (Ref = No)	–**6.22 (**–**8.98**, –**3.47)**	–**3.45 (**–**6.24**, –**0.67)**	–**3.64 (**–**5.96**, –**1.33)**	0.37 (−2.18, 2.91)
Dependence on assistive devices (Ref = No)	–**16.58 (**–**19.29**, –**13.86)**	–**4.77 (**–**7.52**, –**2.01)**	–**8.20 (**–**10.48**, –**5.92)**	–**5.75 (**–**8.27**, –**3.24)**
Social support				
Tangible support	−0.05 (−0.15, 0.05)	−0.02 (−0.12, 0.08)	0.10 (−0.02, 0.18)	−0.02 (−0.11, 0.06)
Emotional/informational support	0.08 (−0.08, 0.24)	**0.29 (0.12, 0.45)**	**0.20 (0.06, 0.33)**	**0.20 (0.04, 0.35)**
Positive social interaction	**0.43 (0.27, 0.58)**	**0.30 (0.15, 0.46)**	**0.23 (0.11, 0.36)**	**0.44 (0.30, 0.58)**
Affectionate support	−0.05 (−0.18, 0.08)	0.06 (−0.07, 0.19)	−0.01 (−0.12, 0.10)	−0.01 (−0.13, 0.11)

Data are presented as coefficients and their 95% confidence intervals. Bold values indicate statistical significance which is defined as p-value <0.05.

**Table 6:** Indirect effects of migration on the four dimensions of QoL for urban adults with rare diseases.

**Mediators**	**Migration status**	**Physical QoL**	**Psychological QoL**	**Environmental QoL**	**Social QoL**
Family number	−0.17 (−0.44, 0.09)	–	–	–	–
Individual income	−4.59 (−13.93, 4.75)	–	–	–	–
CHE	–**0.47 (**–**0.93**, –**0.01)**	0.65 **(**−0.08, 1.48)	0.38 **(**−0.05, 0.98)	0.33 **(**−0.04, 0.85)	–
Tangible support	–**3.70 (**–**6.62**, –**0.79)**	–	–	–	–
Emotional/informational support	0.94 (−0.77, 2.64)	–	–	–	–
Positive social interaction	−1.47 (−3.31, 0.37)	–	–	–	–
Affectionate support	1.65 (−0.49, 3.79)	–	–	–	–

Data are presented as coefficients and their 95% confidence intervals. Column 2 presents the results from “migration status – mediator”, controlling for other variables listed in Table 1. Column 3–6 presents the results from “migration status – mediator – corresponding QoL”, controlling for other variables listed in Table 1. “–” indicates that the potential mediators have no significant associations with the corresponding QoL or migration status has no significant associations with mediators. Bold values indicate statistical significance which is defined as p-value <0.05.

## Discussion

This study firstly assessed the associations of migration with QoL among adults with rare diseases to our best knowledge. Among those who originally lived in rural areas, migration had positive direct associations with physical QoL and environmental QoL, and positive indirect associations with physical QoL and social QoL *via* individual income, as well as a negative indirect association with environmental QoL *via* tangible support. Among those who originally lived in urban areas, neither direct nor indirect associations of migration with QoL were found.

In this study, the proportion of migrants among rural participants with rare diseases was 20.3%, which was slightly higher than that reported among urban participants (15.8%) as well as that reported in the United States (9) (17%) and Europe (10) (16%). This finding was consistent with the situation in China that high-quality health care was primarily located in economically developed cities (16, 17). In our study samples, participants who were younger and had no dependence on assistive devices were more likely to migrate among both rural and urban groups of adults with rare diseases. This finding was consistent with the previous finding on general populations that younger and healthier individuals have more health capital to migrate (11, 15).

Among our samples who originally lived in rural areas, migrants had a higher individual income, while no such difference was found among those who originally lived in urban areas. On the contrary, among those who originally lived in urban areas, migrants had a higher proportion of CHE, while no such difference was found among those who originally lived in rural areas. This could because that even if rural migrants with rare diseases tend to seek better health services, they could have better job opportunities than their counterparts since they may need to receive better education or learn more professional skills in order to live in the urban areas from the very beginning. Whereas for urban migrants, they may lose their original capital resources, such as social capital contributed by their personal social network or family connections, and have to find a new job or to accumulate their social capital from scratch. Another possible explanation is that urban migrants may migrate mainly for the purpose of diagnosis and treatment of the disease, while rural migrants may be for other financial purposes as well. If so, this difference may imply more unmet health needs among urban migrants.

After controlling the confounders that may affect QoL, migration was found to have positive direct associations with physical and environmental QoL among the group of rural participants. The possible explanations of these findings could be that migration could bring people from rural areas more job opportunities and recreational activities that suit them, and may also enhance their accessibility to high-quality healthcare facilities and community-based resources (33). This could also explain to some extent why we also identified a positive indirect association of migration with social QoL *via* individual income. However, we did not identify significant direct associations of migration with psychological and social QoL. This may be because the positive influence brought by migration were offset by its negative outcomes such as social exclusion, discrimination, and disengagement with original social network (12, 13, 34).

The positive indirect association *via* individual income for rural migrants identified in this study suggested a possible intervention for those with rare diseases and still live in the original rural areas, by offering them more job opportunities and hence higher incomes. The negative indirect association between migration status and environmental QoL *via* tangible support could because of the aforementioned disengagement with original social network, and suggested a possible intervention for those with rare diseases and migrate to a new place, by enhancing their connection with people in new communities, including patient organizations for rare diseases at the local level.

Among the group of urban participants, neither direct nor indirect associations of migration with QoL were found. This could arise from the aforementioned complicated pathways through multiple immediate and offsetting factors, with some factors detrimental and others being beneficial (14). Another explanation is that migrants from urban places are more familiar with the living habits in cities and are more likely to adapt to a new urban life easily. Future studies are needed to further explore the mechanism and pathways on how migration affect QoL among urban adults with rare diseases. Nevertheless, the different results indicate the necessity of customized interventions on rural and urban migrants, separately.

This study has some limitations. First, due to the cross-sectional study design, the casual links in this study were inconclusive and needs to be carefully examined in future studies. Second, a non-probability convenience sampling method with a limited number of respondents in this study may lead to biased results, which couldn't properly represent the situation at national level. Third, an online survey was used to collect data. Although this approach could maximize the coverage of rare disease patients as widely as possible, it was possible that the participants were not able to understand the questions correctly or could easily distort the answers. Nonetheless, previous studies showed that the online survey could yield a higher response rate than the mail survey and more accurate results than the telephone survey (35, 36). Hence, it is acceptable to use an online tool to collect information on rare disease patients in the current study. Fourth, although the majority of migrants with rare diseases tends to migrate to urban areas in China, the direction of migration to rural areas may also exist, which should be taken into account separately. Nonetheless, in China, approximately 70% of the migrant population migrate from rural to urban areas (37), thus the direction of migration to urban areas could represent most of the cases among migrants. Lastly, it is possible that the rarer the disease, the more likely that patients would like to migrate to access appropriate care. However, in previous studies that our research team conducted, we found that the rarity of the disease had no significant effect on healthcare utilization across cities among adult rare disease patients in China (16, 17). Hence, we paid more attention to the impact of socioeconomic characteristics of rare disease patients instead of the rarity of the disease in the current study. Future studies are needed to further assess the association between migration status and QoL depending on the disease type among rare disease patients.

## Conclusion

Among rural adults with rare diseases, migration was found to have positive direct effect on physical and environmental QoL, positive indirect effect on physical and social QoL through increased individual income, and negative indirect effect on environmental QoL *via* reduced tangible support. By contrast, neither direct nor indirect associations of migration with QoL were significant among the group of urban participants. The different results may indicate the necessity of customized interventions on rural and urban migrants, separately. In addition, the mechanism and pathways on how migration affect QoL among urban adults with rare diseases need to be further explored in future studies.

## Data availability statement

The original contributions presented in the study are included in the article/supplementary material, further inquiries can be directed to the corresponding author.

## Ethics statement

The studies involving human participants were reviewed and approved by the Committee on the Use of Human and Animal Subjects in Teaching and Research, Hong Kong Baptist University (HASC no.: FRG2/15-16/052). The patients/participants provided their written informed consent to participate in this study.

## Author contributions

DD developed the questionnaire, collected the data, conducted a preliminary analysis, and revised the manuscript. HZ and SC conducted the full data analysis and wrote the initial draft of the manuscript. All authors contributed to the article and approved the submitted version.

## Funding

SC's research was supported by the UK Alzheimer's Society (grant AS-PG-16-006). The funding source had no role in the study design; in the collection, analysis, and interpretation of data; in the writing of the report; and in the decision to submit the article for publication. All authors of the manuscript had full access to all of the data in the study and can take responsibility for the integrity of the data and the accuracy of the data analysis.

## Conflict of interest

The authors declare that the research was conducted in the absence of any commercial or financial relationships that could be construed as a potential conflict of interest.

## Publisher's note

All claims expressed in this article are solely those of the authors and do not necessarily represent those of their affiliated organizations, or those of the publisher, the editors and the reviewers. Any product that may be evaluated in this article, or claim that may be made by its manufacturer, is not guaranteed or endorsed by the publisher.

## Abbreviations

HRQoL: health-related quality of life
QoL: quality of life
CHE: catastrophic health expenditure
MOS-SSS-CM: Medical Outcomes Study Social Support Survey
WHOQOL-BREF: World Health Organization Quality of Life-Brief Version
IQR: interquartile ranges.
